# Effects of Endogenous PPAR Agonist Nitro-Oleic Acid on Metabolic Syndrome in Obese Zucker Rats

**DOI:** 10.1155/2010/601562

**Published:** 2010-07-05

**Authors:** Haiping Wang, Haiying Liu, Zhanjun Jia, Guangju Guan, Tianxin Yang

**Affiliations:** ^1^Division of Nephrology and Hypertension, Department of Internal Medicine, University of Utah, 30 N 1900 E, Rm 4R312, Salt Lake City, UT 84132, USA; ^2^Department of Nephrology, 2nd Affiliated Hospital, Shandong University, Jinan, China

## Abstract

Nitroalkene derivatives of nitro-oleic acid (OA-NO_2_) are endogenous lipid products with novel signaling properties, particularly the activation of PPARs. The goal of this proposal was to examine the therapeutic potential of this OA-NO_2_ in treatment of obesity and obesity-related conditions in obese Zucker rats. The animals were randomly divided to receive OA-NO_2_, oleic acid (OA), both at 7.5 *μ*g/kg/d, or vehicle ethanol via osmotic mini-pumps for 2 weeks. Following OA-NO_2_ treatment, food intake was decreased as early as the first day and this effect appeared to persist throughout the experimental period. At day 14, body weight gain was significantly reduced by OA-NO_2_ treatment. This treatment significantly reduced plasma triglyceride and almost normalized plasma free fatty acid and significantly increased plasma high-density lipid (HDL). The plasma TBARS and proteinuria were paralelly decreased. In contrast, none of these parameters were affected by OA treatment. After 14 days of OA-NO_2_ treatment, hematocrit, a surrogate of fluid retention associated with PPAR*γ* agonists, remained unchanged. Together, these data demonstrated that OA-NO_2_ may offer an effective and safe therapeutic intervention for obesity and obesity-related conditions.

## 1. Introduction

The prevalence of overweight and obesity has dramatically increased during the past two decades. According to results of recent National Health and Nutrition Examination Survey (NHANES), 66.3% of adults in the United States are overweight or obese defined as a body mass index (BMI) of 25 or greater. Of these, 32.2% are obese defined as a BMI of 30 or greater [1]. The World Health Organization has estimated that worldwide, over one billion adults are overweight, with at least 300 million of them being obese [2]. Obesity is associated with a variety of chronic diseases, including endocrine disorders including metabolic syndrome, type 2 diabetes, and dislipidemia; cardiovascular disease including hypertension, congestive cardiomyopathy, stroke, and coronary heart disease; respiratory disorders including dyspnea and obstructive sleep apnea; depression, sleep disorders, musculoskeletal disorders, and gallbladder disease. Life style modification including exercise and calorie restriction remains the cornerstone of anti-obesity therapy but the long term success rate is low. In recent years, pharmacotherapy of obesity has received much attention. 

Recently, nitrated free fatty acids (NO_2_-FA), notably nitroalkene derivatives of linoleic acid (nitrolinoleic acid, LNO_2_) and oleic acid (OA-NO_2_) are found to be endogenous molecules with several attractive signaling properties [3, 4]. In particular, nitroalkenes are found to be a robust endogenous ligand for peroxisome proliferator-activated receptor *γ* (PPAR*γ*) and they also activate PPAR*α* and PPAR*δ* at increasing concentrations [3, 4]. PPARs have emerged as a novel therapeutic target for treatment of various components of metabolic syndrome. For example, synthetic PPAR*γ* agonists, thiazolidinediones, have been widely used as insulin sensitizing agents for treatment of type 2 diabetes; synthetic PPAR*α* agonists such as clofibrate and fenofibrate have been used in clinics for more than 30 years as lipid lowering agents [5–8]. Unfortunately, these synthetic agents are often associated with various toxicities [7, 9–11]. The goal of the present study was to examine the therapeutic potential of OA-NO_2_ in an experimental model of obesity and metabolic syndrome.

## 2. Materials and Methods

### 2.1. Animals

Four month-old male obese Zucker and lean rats were purchased from Charles River Laboratory (Wilmington, MA). All animals were housed at the University of Utah Comparative Medicine Center, maintained on a 12-hour light/dark cycle, and provided food and water ad libitum. Procedures and protocols followed guidelines set by the Laboratory Animal Care Committee at the University of Utah.

### 2.2. Materials

9-Nitrooleic acid and 10-nitrooleic acid are two regioisomers of nitrooleic acid (OA-NO_2_). These regioisomers are formed by nitration of oleic acid in approximately equal proportions as described elsewhere [4]. The two compounds were purchased from Cayman Chemicals (Ann Arbor, MI) (9-nitrooleic acid: Cat#10008042; 10-nitrooleic acid: Cat#10008043) and used as a 1:1 mixture of the isomers. 

### 2.3. Protocols for Animal Experiments

Under general anesthesia, obese Zucker rats were subcutaneously implanted with a mini-pump (DURECT Corporation, Cupertino, CA) delivering OA-NO_2,_ oleic acid (OA), both at 7.5 *μ*g/kg/d, or vehicle ethanol for 2 weeks. Age and gender matched lean rats with vehicle treatment were used as controls. Food intake was determined periodically; body weight and collections of blood and 24-h urine samples were determined at the end of the experiments. Animals were fasted from 6:00 pm to 9:00 am before blood sampling. Blood sampling was conducted by making a small cut (~2 mm) in the tail using razor blade. 24-hour urine was collected using metabolic cages.

### 2.4. Plasma Glucose and Lipids

The following determinations were analyzed by using ROCHE, Modular P in the University Hospital Chemistry Laboratory: glucose, cholesterol, triglyceride, HDL, and LDL; non-esterided fatty acids were determined by using a colorimetric method (Cat# SFA-5, ZenBio, Research Triangle Park, NC).

### 2.5. Hematocrit

Hematocrit was determined as the popular ways. Briefly, 5–10 *μ*l of blood was collected from tail cut using a 10 *μ*l capillary glass (Idaho Technology). One side of the tube was sealed with Hemato-Seal and then centrifuged for 4 minutes in a Thermo IEC microcentrifuge machine. The total height of sample and height of the red blood cell column were measured. The hematocrit reflects the ratio between the red blood cell column and total height.

### 2.6. Measurement of Thiobarbituric Acid-Reactive Substances (TBARS)

The measurement of TBARS was based on the formation of malondialdehyde (MDA) by using a commercially available TBARS Assay kit (Cat#: 10009055, Cayman Chemical, Ann Arbor, MI).

### 2.7. Total Urine Protein

24-h urinary protein excretion was measured by using Commassie blue.

### 2.8. Statistical Analyses

All data are presented as means ± SEM. Differences between groups were analyzed by unpaired *t* test or 2-way ANOVA, followed by Bonferroni post hoc test using GraphPad Prism software version 3.03 (GraphPad Software, San Diego, CA). Differences within groups, before and after OA-NO_2_ treatment, were analyzed by paired *t* test. A probability value of < 0.05 was considered significant.

## 3. Results

### 3.1. Effect of OA-NO_2_ on Food Intake and Body Weight Gain

**Figure 1:**
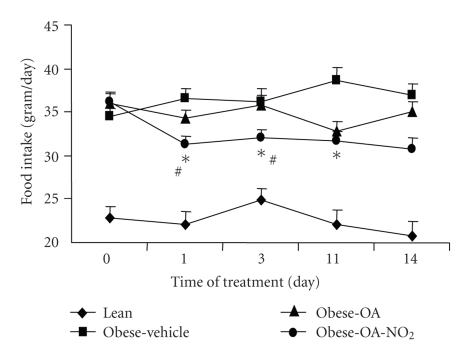
Food intake in male 4-mo-old obese Zucker rats (obese) over 14 days of infusion with vehicle, OA-NO_2_, or OA, each at 7.5 *μ*g/kg/d via osmotic mini-pumps. Age and gender matched lean rats with vehicle treatment were used as controls. Lean: *n* = 3; Vehicle: *n* = 7; OA-NO_2_: *n* = 5; OA: *n* = 5. *, *P* < .01 versus Obese/Vehicle; ^#^, *P* < .05 versus OA during the corresponding period. Data are mean ± SE.

Starting from 4 months of age, the obese Zucker rats were chronically infused for 2 weeks with vehicle, OA-NO_2_ or OA each at 7.5 *μ*g/kg/day via an osmotic mini-pump. Untreated age-matched lean rats were used as controls. As shown in Figure 1, OA-NO_2_ treatment in obese Zucker rats reduced food intake as early as day 1 (Vehicle 36.6 ± 1.01 versus OA-NO_2_ 31.2 ± 1.04 g, *P* < .01), and this effect appeared to persist throughout the experiment except that only a trend was detected on day 14. Over the 14-day period, obese Zucker rats exhibited a significant increase in body weight as compared with lean controls (obese Zucker 48.0 ± 4.0 versus lean 16.7 ± 1.2 g, *P* < .01). The body weight gain in obese Zucker rats was reduced to 34.0 ± 1.1 g by OA-NO_2_ treatment (*P* < .05). In contrast, a 14-day treatment with OA in obese Zucker rats at the same dose and same infusion rate did not affect food intake or body weight.

### 3.2. Effect of OA-NO_2_ on Biochemical Parameters

At baseline, obese Zucker rats already developed significant hypertriglyceridemia as compared with lean controls (549.1 ± 63.4 versus 78.0 ± 11.2 mg/dl, *P* < .01) and the values continued to rise on day 14. The baseline values of plasma triglyceride in the OA-NO_2_ and OA groups (OA-NO_2_: 556.8 ± 45.8 mg/dl; OA: 637.0 ± 102.0 mg/dl) were comparable to the vehicle group. At day 14, the values in OA-NO_2_ group (423.0 ± 46.0 mg/dl) but not OA group (687.0 ± 99.0 mg/dl) was significantly lower than the vehicle control (877.4 ± 64.9 mg/dl). Plasma non-esteried free fatty acids followed a similar pattern as plasma triglyceride. Of particular note, a 14-day treatment with OA-NO_2_ but not OA decreased plasma non-esteried free fatty acid concentrations to a value almost comparable to the lean control. In contrast, plasma cholesterol was unaffected by OA-NO_2_ or OA. Interestingly, a 14-day OA-NO_2_ treatment elevated plasma HDL from 26.8 ± 1.49 to 32.6 ± 2.6 mg/dl (*P* < .05), paralleled by the increased HDL to total cholesterol ratio. In contrast, both plasma HDL and the HDL to cholesterol ratio remained unchanged after 14-day treatment with vehicle and OA. At day 14, obese Zucker rats exhibited normal glycemia (93.7 ± 6.9 versus mg/dl) that was not affected by OA-NO_2_ treatment (85.6 ± 5.3 mg/dl, *P* > .05). Plasma aspartate aminotransferase (AST)/alanine aminotransferase (ALT) and BUN were evaluated to reflect liver and kidney function, respectively; none of these parameters were affected by OA-NO_2_ treatment (AST: 156.8 ± 18.4 versus 159 ± 7.7 U/L, *P* > .05; ALT: 135.0 ± 18.9 versus 104.5 ± 3.9 U/L, *P* > .05; BUN: 15.0 ± 1.1 versus 15.8 ± 0.7 mg/dl, *n* = 4-5, *P* > .05). Therefore, these data do not indicate obvious hepatic or renal toxicities associated with OA-NO_2_.

### 3.3. Effects of OA-NO_2_ on Oxidative Stress, Proteinuria, and Hematocrit

Plasma TBARS was assessed as a measure of oxidative stress in obese Zucker rats. The obese rats had a marked increase in plasma TBARS as compared with lean controls (14.83 ± 1.47 versus 1.97 ± 0.63 *μ*M, *P* < .01). A 14-day OA-NO_2_ treatment decreased plasma TBARS levels to 8.26 ± 2.31 *μ*M (*P* < .01 versus the baseline in the same group or the vehicle group at the corresponding time period). As compared with the lean control (12.22 ± 0.73 mg/24 hours), obese Zucker rats exhibited progressive proteinuria (day 0: 34.88 ± 8.17; day 14: 78.5 ± 19.7 mg/24 hours). OA treatment tended to exhibit an anti-proteuric effect but this did not reach a statistical significance. 

PPAR*γ* agonists are limited by significant fluid retention as reflected by body weight gain and plasma volume expansion. The drop of hematocrit has been used as a surrogate marker of plasma volume expansion since these compounds do not influence erythropoiesis. Therefore we examined hematocrit in awake animals; hematocrit remained unchanged irrespective of OA-NO_2_ or OA treatment.

## 4. Discussion

linoleic acid (nitrolinoleic acid, LNO_2_) and oleic acid (OA-NO_2_) are two major nitroalkene derivatives formed endogenously via NO-dependent oxidative reactions [12, 13]. Studies in cell cultures have demonstrated PPAR ligand activities of these derivatives [3, 4]. In this regard, LNO_2_ was initially reported to be a potent an endogenous ligand for PPAR*γ* that acts within physiological concentration ranges [3]. Subsequently, OA-NO_2_ was shown to be a more robust PPAR*γ* activator as compared with LNO_2_ and it also activated PPAR*α* and PPAR*δ* at increasing concentrations [3, 4]. The three subtypes of PPARs are critically important for the control of glucose homeostasis and lipid metabolism. Therefore we were prompted to evaluate the therapeutic potential of nitroalkene derivatives in an animal model of obesity and metabolic syndrome. Here we demonstrate that a 14-day osmotic delivery of a low dose (7.5 *μ*g/kg/d) of OA-NO_2_ in obese Zucker rats produced beneficial effects on various components of metabolic syndrome, including obesity, hyperlipidemia, and proteinuria, accompanied by an immediate reduction of food intake. 

Epidemiological studies demonstrate that ingestion of polyunsaturated fatty acids (PUFA) and monounsaturated fatty acids (MUFA) but ingestion of saturated fatty acid deteriorates insulin resistance and obesity-related diseases [14]. An issue arises as to whether the beneficial effects of OA-NO_2_ observed in obese Zucker rats are attributable to the native OA. This possibility is not supported by a previous study showing that chronic administration of dietary oleic acid in obese Zucker at a dose roughly 500 times higher than that in the present study fails to affect body weight, plasma glucose and lipids despite the amelioration of pancreatic islet disruption [15]. Along this line, the benefits of omega-3 polyunsaturated fatty acid (LC omega-3 PUFA) are also seen with a substantially high dose [16]. More importantly, we found that administration of OA at the same dose and with the same route did not produce any measurable effects on body weight or other metabolic parameters. It appears evident that the beneficial effects of OA-NO_2_ observed in obese Zucker rats are derived from the nitration of OA but not the native fatty acid. 

The mechanism of action of OA-NO_2_ in obese Zucker rats remains elusive but the involvement of PPARs appears conceivable. PPAR*α* agonists (Wy-14643 and GW7647) and endogenous PPAR*α* agonists (oleylethanolamide;OEA) treatment induces satiety and reduces body weight gain [17–20], an effect almost analogous to that of OA-NO_2_ in obese Zucker rats. The rapid onset of appetite-suppressing effect (within a day) between PPAR*α* agonists and OA-NO_2_ is also similar. Furthermore, PPAR*α* agonists are well known to reduce plasma triglyceride and increase HDL, a profile again similar to that of OA-NO_2_. Together, PPAR*α* activity appears to explain most of metabolic effects of OA-NO_2_ in obese Zucker rats. Of note, both OEA and OA-NO_2_ are naturally occurring products derived from the same precursor, OA, and exert a similar role in regulation of satiety and body weight. It is an intriguing possibility that the two derivatives of OA converge on a common final pathway involving PPAR*α*. However, the differences may exist in regulation of their production. OEA is produced by the small intestine in response to food intake [17, 20] while the production of OA-NO_2_ appears to be primarily regulated by NO-dependent oxidative reactions which may or may not be related to feeding. 

Although OA-NO_2_ is a robust PPAR*γ* activator in an in vitro system, the involvement of PPAR*γ* in OA-NO_2_ signaling in the current experimental model might be minimal. In a sharp contrast to the appetite-suppressing and weight-lowering effects of OA-NO_2_, the synthetic PPAR*γ* agonists, thiazolidinediones, cause increases in food intake [21, 22] and body weight and plasma volume expansion [10, 23, 24]. The lack of an effect of the PPAR*δ*/*β* agonist on food intake [17] also does not support involvement of PPAR*δ* in the anorectic action of OA-NO_2_. However, it is still possible that these two PPAR subtypes may partially contribute to some aspects of OA-NO_2_ action, such as the lipid-lowering and proteinuria-lowering effects. Indeed, all three PPAR subtypes share common lipid-lowering properties [25–28], and PPAR*α* [29–32] and PPAR*γ* [33–36] exert similar renoprotective effects in animals or humans with diabetic nephropathy. It should be pointed out that in addition to PPARs, nitrated lipids can engage other pathways such as NO, NF-kB, and hemoxygnease-1. Whether these PPAR-independent or other as yet unidentified pathways play a contributory role in mediating the metabolic actions of OA-NO_2_ remains unclear. 

Whatever the underlying mechanism, our data suggest that OA-NO_2_ may offer therapeutic opportunities for management of obesity and obesity-related conditions. At the present, most of the anti-obesity agents target neurotransmitter receptors and are often associated with increased risk of psychiatric events. One example is rimonabant, a selective antagonist of the cannabinoid type 1 receptor, which is the most officious weight-lowering agent but causes depression and anxiety [37–39]. Because of the psychiatric side effects, the Committee for Medicinal Products for Human Use of the European Medicines Agency (EMEA) has recommended suspension of rimonabant as an anti-obesity therapy [40]. OA-NO_2_ is unique in that it is a naturally occurring product likely acting nuclear receptors, thus representing a novel class of weight-lowering agents. We demonstrated that it is not only highly effective but also well tolerated. Moreover, OA-NO_2_ exhibited beneficial effects beyond weight loss. For example, it improved lipid profile, suppressed oxidative stress, and reduced proteinuria. 

In summary, the present study describes novel therapeutic potential of OA-NO_2_ in a rodent model of obesity and metabolic syndrome. A low-dose treatment produces anorexia and reduces body weight gain, accompanied by improved lipid profile, and reduction of oxidative stress and proteinuria, in the absence of plasma volume expansion. These effects are derived from the nitration of OA rather than the native fatty acid. As a natural product, OA-NO_2_ appears to be an optimal pharmacotherapy for obesity and obesity-related conditions with a superior efficacy and safety profile. 

## Figures and Tables

**Figure 2:**
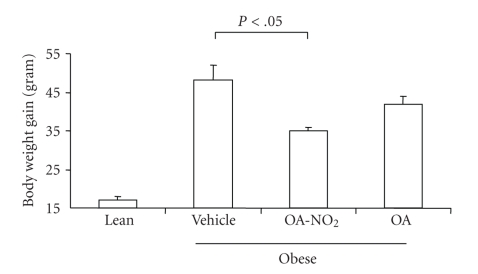
Body weight gain in obese Zucker rats before and after 14-d infusion with vehicle, OA-NO_2_, or OA. Lean rats with vehicle treatment were used as controls. Lean: *n* = 3; Vehicle: *n* = 7; OA-NO_2_: *n* = 5; OA: *n* = 5. Data are mean ± SE.

**Figure 3:**
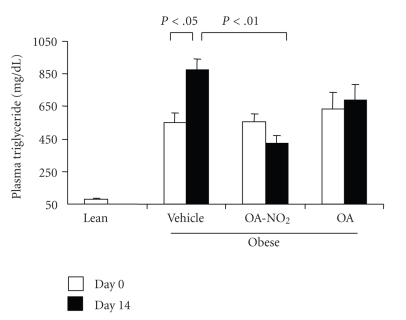
Plasma triglyceride in obese Zucker rats before and after 14-d infusion with vehicle, OA-NO_2_, or OA. Lean rats with vehicle treatment were used as controls. Lean: *n* = 3; Vehicle: *n* = 7; OA-NO_2_: *n* = 5; OA: *n* = 5. Data are mean ± SE.

**Figure 4:**
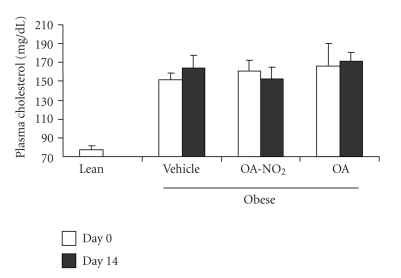
Plasma cholesterol in obese Zucker rats before and after 14-d infusion with vehicle, OA-NO_2_, or OA. Lean rats with vehicle treatment were used as controls. Lean: *n* = 3; Vehicle: *n* = 7; OA-NO_2_: *n* = 5; OA: *n* = 5. Data are mean ± SE.

**Figure 5:**
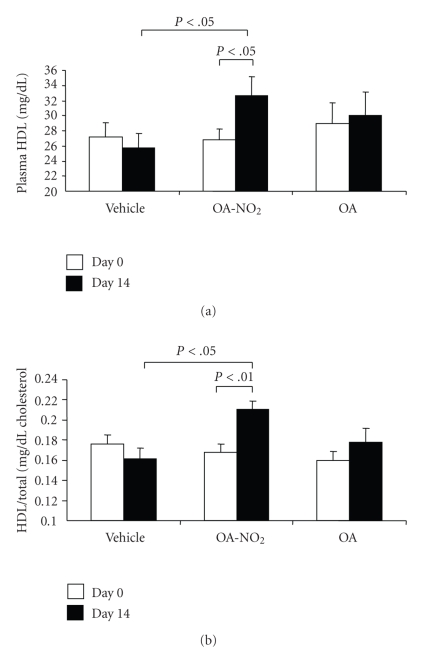
Plasma HDL (a) and the ratio of HDL to total cholesterol (b) before and after 14-d infusion with vehicle, OA-NO_2_, or OA. Lean rats with vehicle treatment were used as controls. Lean: *n* = 3; Vehicle: *n* = 7; OA-NO_2_: *n* = 5; OA: *n* = 5. Data are mean ± SE.

**Figure 6:**
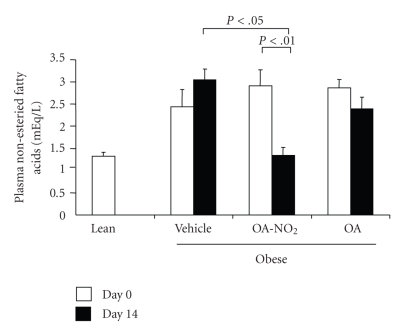
Plasma non-esteried fatty acids in obese Zucker rats before and after 14-d infusion with vehicle, OA-NO_2_, or OA. Lean rats with vehicle treatment were used as controls. Lean: *n* = 3; Vehicle: *n* = 7; OA-NO_2_: *n* = 5; OA: *n* = 5. Data are mean ± SE.

**Figure 7:**
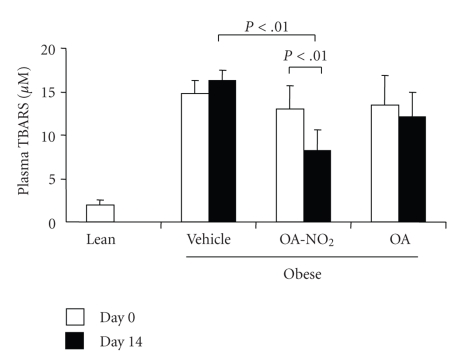
Plasma TBARS in obese Zucker rats before and after 14-d infusion with vehicle, OA-NO_2_, or OA. Lean rats with vehicle treatment were used as controls. Lean: *n* = 3; Vehicle: *n* = 7; OA-NO_2_: *n* = 5; OA: *n* = 5. Data are mean ± SE.

**Figure 8:**
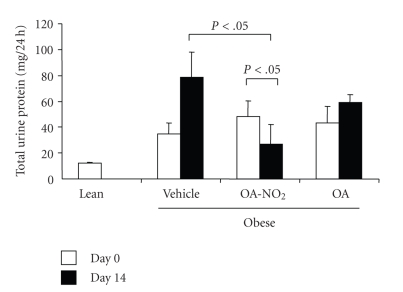
Proteinuria in obese Zucker rats before and after 14-d infusion with vehicle, OA-NO_2_, or OA. Lean rats with vehicle treatment were used as controls. Lean: *n* = 3; Vehicle: *n* = 7; OA-NO_2_: *n* = 5; OA: *n* = 5. Data are mean ± SE.

**Figure 9:**
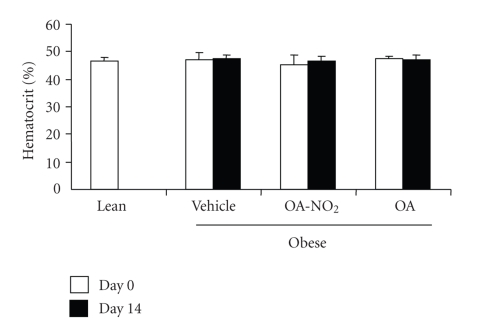
Hematocrit in obese Zucker rats before and after 14-d infusion with vehicle, OA-NO_2_, or OA. Lean rats with vehicle treatment were used as controls. Lean: *n* = 3; Vehicle: *n* = 7; OA-NO_2_: *n* = 5; OA: *n* = 5. Data are mean ± SE.
